# Diagnostic performance of a dual *western blot* for the simultaneous detection of anti-HTLV-1 and anti-HIV-1 antibodies

**DOI:** 10.17843/rpmesp.2026.433.16741

**Published:** 2026-09-28

**Authors:** Eduardo Miranda-Ulloa, Soledad Romero-Ruiz, Ronal Briceño-Espinoza, Andrés Vásquez-Domínguez, Dilan Suárez-Agüero

**Affiliations:** 1 Laboratorio de Referencia Nacional de Virus de Transmisión Sexual, Centro Nacional de Salud Pública, Instituto Nacional de Salud, Lima, Peru. Laboratorio de Referencia Nacional de Virus de Transmisión Sexual Centro Nacional de Salud Pública Instituto Nacional de Salud Lima Peru

**Keywords:** Western Blotting, HTLV-1, HIV-1, Diagnosis, Sensitivity and Specificity

## Abstract

This research aimed to determine the performance of an *in house* dual *western blot* (WB) for the individual and simultaneous detection of anti-HTLV-1 and anti-HIV-1 antibodies; the strip was designed with HTLV-1 antigen (1.67 ug/mm) on top and HIV-1 (1.34 ug/mm) on the bottom. The *in-house* dual WB was evaluated using 260 reference serum samples from a serum bank: 50 HTLV-1/HIV-1 positive, 50 HIV-1 positive, 60 HTLV-1 positive, 50 negative, and 50 potentially interfering samples. Accuracy was evaluated in five regions of Peru. Overall sensitivity and specificity were 96.3% (95% CI: 93.0‒99.5) and 100% (95% CI: 99.5‒100.0), respectively. Individually, sensitivity was 94% in HTLV-1/HIV-1 coinfected individuals, 96.7% in HTLV-1, and 98% in HIV-1. Repeatability and reproducibility were 100% under conditions of 34‒3256 m a.s.l., 16.4‒37.9 °C, and 42‒95% relative humidity. We conclude that the *in house* dual HTLV-1/HIV-1 WB shows high diagnostic performance.

## INTRODUCTION

Human T-lymphotropic virus type 1 (HTLV-1) and human immunodeficiency virus type 1 (HIV-1) are the most prevalent retroviruses worldwide ^1^^,^^2^. The World Health Organization (WHO) recognizes critical epidemiological gaps concerning HTLV-1, although previous studies estimated that in Peru there are at least 450,000 people infected with HTLV-1. WHO emphasizes the urgent need to implement active epidemiological surveillance to ascertain the true global burden of the disease ^1^; meanwhile, between 2021-2025, the Ministry of Health (MINSA) of Peru reported 46,975 new cases of HIV-1 ^3^.

In Peru, people living with HIV receive free monitoring and antiretroviral treatment, minimizing the risk of sexual and vertical transmission ^4^, while people carrying HTLV-1 do not enjoy that benefit, due to the lack of systematic screening and clinical follow-up programs. Implementing a similar strategy in Peru is an urgent need, since, given the lack of an effective antiretroviral treatment, maternal screening and the subsequent regulation of breastfeeding constitute the only effective window of opportunity to break the chain of intergenerational transmission of this high-cost and socially impactful disease ^1^.

There are investigations that have described prevalences of 36.4% (1250/3431) of HTLV-1 in relatives of index cases ^5^ and of the severe clinical impact reflected in a Peruvian hospital that diagnosed cancer in 1934 virus carriers ^6^. Like other countries, Peru prevents the blood transmission of HTLV-1 in its blood banks ^1^, but only Brazil ^7^ and Chile ^8^ screen their pregnant and postpartum women in prenatal care to prevent mother-to-child transmission ^7^^,^^8^, whose rate ranges from 3.9% to 27% ^1^.

HTLV-1 and HIV-1 have similar risk factors and shared transmission routes. In the Peruvian population, it has been reported that between 10% and 18.6% of people infected with HIV-1 present coinfection with HTLV-1 ^9^^,^^10^. This coinfection has been associated with a higher risk of death and lower survival compared to infection solely by HIV-1, which underlines the importance of HTLV-1 diagnosis in people with HIV-1 ^11^.

In serological diagnosis, *immunoblot* constitutes the reference assay for HIV and HTLV confirmation. Currently, tests are commercialized independently for each pathogen ^12^^,^^13^, which leads to performing multiple assays when coinfections need to be evaluated ^9^^-^^11^, increasing time, costs, and operational complexity. In this context, to our knowledge, to date, there is no international commercial system that allows simultaneous confirmation of both retroviruses. Given this, the proposed innovation consists of the development of a dual diagnostic *western blot* test based on a strip containing, in immobilized form, immunogenic antigens for HIV-1 and HTLV-1. The use of this single strip will allow simultaneous confirmation and differentiation of both pathogens. The development of a dual diagnostic test could contribute to establishing new diagnostic algorithms, reduce operational costs, and facilitate epidemiological surveillance of both retroviruses in key populations and other risk groups.

As described, the present study aimed to determine the diagnostic performance of a dual *in house western blot* (WB) for the individual and simultaneous detection of anti-HTLV-1 and anti-HIV-1 antibodies.

**KEY MESSAGES**

Motivation for the study. HTLV-1 is an endemic and fatal infection in Peru. Coinfection with HIV-1 increases the risk of death, which is why it is necessary to develop dual diagnostic tools for both retroviruses.

Main findings. Overall sensitivity and specificity of 96.3% and 100% respectively. Individually, sensitivity was 94% in coinfected individuals, 96.7% in HTLV-1, and 98% in HIV-1, without false negatives, but indeterminate results were included in the sensitivity calculations. Repeatability and reproducibility were 100% under conditions of different temperature, humidity, and altitude ranges.

Implications. The dual *western blot* is high-throughput, reduces times and costs compared to individual kits, maintaining its efficacy. Due to its low price, it is an ideal alternative for diagnostic confirmation in HTLV-1 and HIV-1 surveillance.

## THE STUDY

A retrospective cross-sectional diagnostic test evaluation design was applied during 2024-2025. The sample size was calculated with an independent estimation of sensitivity (expected: 90%) and specificity (expected: 95%), applying the proportions formula (95% confidence, Z=1.96; admissible error of 5%, W=0.05), being the minimum calculated sizes 139 positive and 73 negative samples.

Non-probabilistic convenience sampling was performed from the Serum bank of the National Institute of Health of Peru, consolidating a final sample of 260 (160 positive and 100 negative) which amply exceeded the minimum required limits.

Following the STARD guideline, a validation design structured in five groups of serum samples was implemented. This strategy allowed for the evaluation of dual and individual sensitivity using groups of samples positive for HTLV-1/HIV-1, HTLV-1, and HIV-1, as well as specificity, using groups of samples from apparently healthy individuals and samples with interferents. Inclusion criteria required a minimum serum volume of 600 µL and the absence of macroscopic evidence of hemolysis, lipemia, or microbial contamination. Conversely, samples with insufficient volume, without an identification code, or lacking a previous result that would allow for the classification of their condition were excluded.

The distribution of the 260 samples into five sample groups consisted of: HTLV-1/HIV-1 coinfected positive (n_1_=50); HIV-1 positive (n_2_=50); HTLV-1 positive (n_3_=60); HTLV-1/2 negative from apparently healthy people (n_4_=50); interfering (n_5_=50), with other conditions and infections: dengue (5), hepatitis B (5), *Strongyloides* (5), tuberculosis (5), uveitis (5), thyroiditis (5), pregnant women (5), rheumatoid disease (5), lymphoma (5) and spastic paraplegia (5).

Serological characterization was performed using as reference standard (Gold Standard) the commercial Immunoblot INNO-LIA™ HTLV I/II ^12^ and INNO-LIA™ HIV-1/2 ^13^^)^ tests from the Fujirebio brand, manufactured in Belgium, which are globally validated confirmation methodologies. The 260 serum samples were processed by both reference tests.

To ensure the blinding of the tests in the five groups, the samples were randomly coded before being processed with the two reference standards. Subsequently to obtaining their results, those that met the established criteria for each group were selected.

The interferent group was included for the purpose of evaluating cross-reactivity and general analytical interference, both being addressed jointly, and although the sample size was limited to five samples per sub-condition, which restricts individual statistical analysis by subgroups, the consolidated sample size (n_5_=50) provided adequate statistical power.

The samples from apparently healthy individuals were originally collected from blood bank donors who tested negative in screening tests for the seven mandatory markers: HIV-1/2, HTLV-1/2, HBV (surface antigen and total core antibody), HCV, Chagas, and syphilis.

For the preparation of HTLV-1 and HIV-1 antigens, MT-2+HTLV-1 and H9+HIVIIIB cell lines were cultured *in vitro*, following the procedure described by Romero-Ruiz *et al.*^14^; HTLV-1 cell pellet concentrations ranged from 30,050,000 to 41,000,000 cells, and for HIV-1, they ranged from 25,250,000 to 45,350,000 cells. Each cell pellet was washed, centrifuged, lysed, quantified, and the antigens were treated according to the procedure detailed by Miranda-Ulloa *et al*^15^; the HTLV-1 protein concentration range was between 0.31 to 0.56 ug/ul, and for HIV-1, between 0.23 to 0.52 ug/ul. Four batches of antigens from each virus were prepared, and their proteins were characterized in polyacrylamide gels and stained with Coomassie blue (supplementary material figures S1 and S2).

The multiple WB for parasites developed by Davelois *et al.*^16^^)^ boosted the creation of the dual methodology, taking as a starting point the individual methodologies developed by our research team ^15^^,^^17^ with certain modifications, finally, the standardized process was carried out with the following steps:

The treated antigens of each virus were individually subjected to electrophoresis on different gels, using 8.3 × 7 × 0.1 cm mini-gels from the Mini PROTEAN® Tetra Cell-Bio Rad Laboratories, with a 15% resolution gel and a 10% stacking gel. The gel system had three lanes, one 60 mm and the others 5 mm, located at the extreme sides. The antigens were loaded into the 60 mm lane with 1.67 ug/mm for HTLV-1 and 1.34 ug/mm for HIV-1. In the extreme side lanes, 2 µL of pre-stained marker per lane was spread. Subsequent steps were performed according to the methodology established by Miranda-Ulloa *et al.*^15^.

A wet electrotransfer was performed in the Trans Blot Transfer Cell; the design consisted of placing the HTLV-1 gel on the upper part of the 0.22 µm nitrocellulose membrane, and the HIV-1 gel on the lower part, previously selecting the specific antigens with the help of the pre-stained marker, which facilitated delimiting the zones of the gels to be cut. The subsequent steps up to membrane blocking and cutting into strips were performed according to the procedure detailed by Miranda-Ulloa *et al.*^15^.

Runs were performed for the five groups of serum samples using eight-channel plates, with the optimal volume for all steps being 1 ml; each strip was immersed in PBS-T-L containing the study sera and controls at a 1/20 dilution for 3 hours under agitation at 90 rpm. The subsequent steps were performed according to the procedure detailed by Miranda-Ulloa *et al.*^15^.

A positive and a negative control were included in each run, which consisted of sera characterized by reference tests and validated by the total presence or absence of diagnostic bands for HTLV-1/HIV-1, respectively.

The reading criteria for the dual *in house* WB were performed as follows: criterion, WHO: positive HTLV-1: reactivity with at least one Env protein (gp46 or gp21) and at least one core protein (p19 or p24) ^18^. Criterion, Serology Network Consortium: positive HIV-1: reactivity with Env protein gp41 and at least one gag or pol protein, p55, p39, p31, p24 or p17 ^19^. Negative: without any specific band. Indeterminate: presence of a single band, or incomplete patterns.

With the results of the five sample groups, the diagnostic accuracy of the dual *in house* WB was determined. Indeterminate results were considered non-positive (negative) in the contingency table when calculating the respective sensitivities. The data from the reference test and the dual *in house* WB were consolidated in Microsoft Excel®. Diagnostic validity was evaluated in Epidat v3.1 (simple diagnostic tests module) using 2x2 contingency tables. Sensitivity, specificity, validity index, PPV, NPV, Youden's index, and likelihood ratios (positive and negative) were calculated, all with 95% confidence intervals (95% CI). The concordance (%) of diagnostic accuracy was evaluated using five reference sera (one serum from each sample group); to determine repeatability, these sera were analyzed in quintuplicate in a single intralaboratory run, and reproducibility was calculated by analyzing them in triplicate on three different days in five distinct interlaboratory geographical settings (La Libertad, Junín, Apurímac, San Martín and Amazonas). This design allowed verifying the performance of the test under diverse environmental conditions of temperature, humidity, and altitude, a critical factor to ensure its viability in a decentralization scenario.

The sera used were anonymized. The study was approved by the Institutional Ethics Committee for Research of the National Institute of Health (OI-012-19) and funded by PROCIENCIA - CONCYTEC (contract N° 022-2019-FONDECYT).

## FINDINGS

The 260 samples were considered in the study and none were excluded, the flowchart presents the distribution of the incorporated sera according to the reference serological status versus the results obtained by the dual *in house* HTLV-1/HIV-1 WB (figure 1).

**Figure 1:**
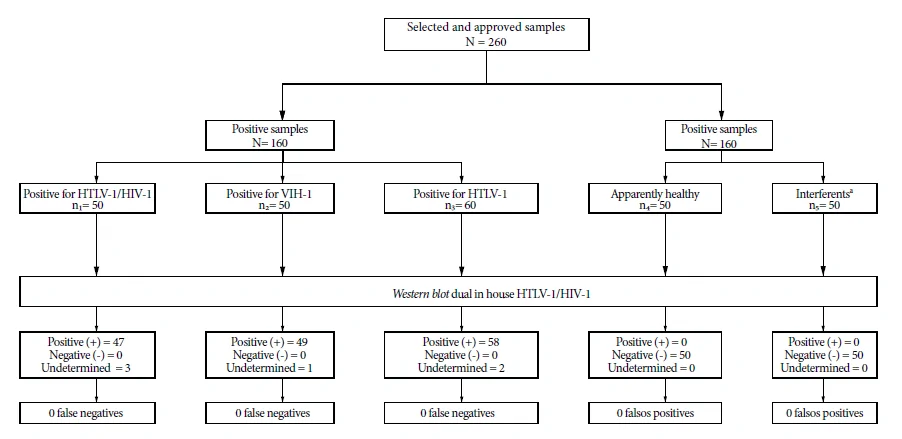
Flowchart of selection and evaluation of the samples included in the study.

Interfering factors: negative for HTLV-1/HIV-1, but with other infections and conditions; dengue (5), hepatitis B (5), Strongyloides (5), tuberculosis (5), uveitis (5), thyroiditis (5), pregnant women (5), rheumatoid disease (5), lymphoma (5) and spastic paraplegia (5).

A dual WB was developed that detects anti-IgG antibodies for HTLV-1 and HIV-1 simultaneously on a single strip. On the strips corresponding to the dual and individual positive controls, the characteristic bands of both retroviruses are visualized accordingly (HTLV-1=p19, gp21, p24, gp46; HIV-1=p17, p24, p31, p39, gp41, p55), while the negative control showed no reactivity against any of the antigenic systems, validating the format used (figure 2).

**Figure 2:**
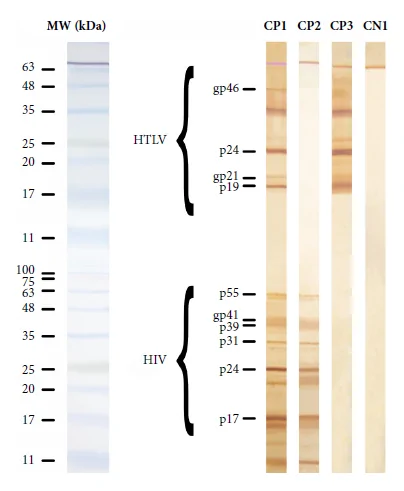
Reactivity pattern of the *western blot* dual in house HTLV-1/HIV-1 test for antibody detection in serum in internal assay controls. CP1: HTLV-1 and HIV-1 positive; CP2: HIV-1 positive; CP3: HTLV-1 positive; CN: negative control.

MW: molecular weight, KD: kilodaltons.

For the evaluation of diagnostic accuracy, the individual sensitivity of the dual *in house* WB was determined with the readings from each positive panel. Thus, individually, a sensitivity of 94.0% (47/50) was obtained with the HTLV-1/HIV-1 coinfected panel (figure 3A), 98.0% (49/50) with the HIV-1 panel (figure 3B), and 96.7% (58/60) with the HTLV-1 panel (figure 3C). Three, one, and two indeterminate results were obtained in each of these three positive panels, respectively (figure 1). The individual specificity was 100% (50/50) with the negative panel (figure 3D) and 100% (50/50) with the interfering panel (figure 3E) (supplementary material table S1).

**Figure 3:**
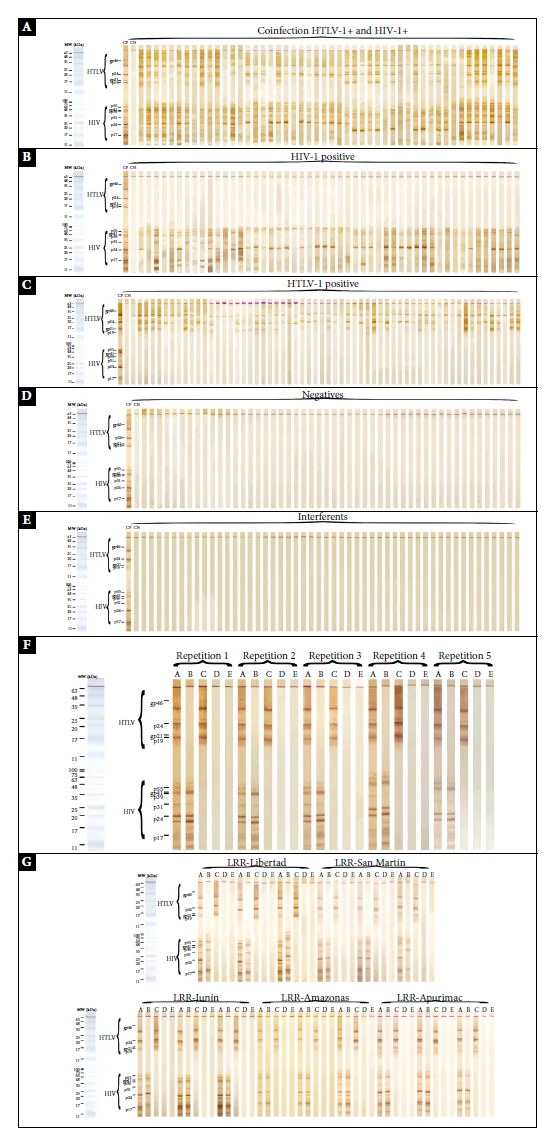
3. Band profiles and evaluation of the diagnostic accuracy and precision of the in-house dual *western blot* HTLV-1/HIV-1. The serum sample groups for diagnostic accuracy included: A) HTLV-1/HIV-1 coinfection positive, B) HIV-1 positive, C) HTLV-1 positive, D) negative, E) interfering. The assay precision was evaluated using the parameters of F) repeatability and G) reproducibility.

When jointly analyzing the three positive panels, an overall sensitivity of 96.3% (154/160) (95% CI: 93.0-99.5) was obtained, while, by grouping the negative and interfering panel, an overall specificity of 100% (100/100) (95% CI: 99.5-100.0) was obtained. Overall, a validity index of 97.7% was obtained, with positive and negative predictive values of 100% and 94.3%, respectively. Likewise, Youden's index was 0.96 and the negative likelihood ratio 0.04 (table 1).

**Table 1:** Evaluation of the diagnostic performance of the *western blot* dual *in house* HTLV-1/HIV-1. General parameters of diagnostic accuracy of the assay, with their 95% confidence intervals (95% CI).

Parameters	***Western blot* dual *in house* HTLV-1/HIV-1**		
	Value	(95% CI)	
Sensitivity (%)	96.3	93.0	99.5
Specificity (%)	100	99.5	100
Validity Index (%)	97.7	95.7	99.7
Positive predictive value (%)	100	99.7	100
Predictive value - (%)	94.3	89.5	99.2
Youden Index	0.96	0.93	0.99
Likelihood ratio +	-	-	
Likelihood ratio -	0.04	0.02	0.08

Regarding the evaluation of diagnostic accuracy, the analysis of the five samples processed by the dual *in house* WB in a single run in quintuplicate under identical conditions showed concordant band profiles, without variations in intensity or in the presence of diagnostic bands, achieving 100% agreement in intralaboratory repeatability (figure 3F). Similarly, the five samples analyzed in triplicate in five reference laboratories with different altitudes of 34, 353, 420, 2378 and 3,256 m a.s.l., within a temperature range of 16.4 °C and 37.9 °C and at an environmental relative humidity between 42% and 95% (supplementary material figure S3), yielded concordant results, achieving 100% interlaboratory reproducibility (figure 3G).

## DISCUSSION

Fujirebio immunoblot reference tests (INNO-LIA) for the detection of anti-HTLV-1 and anti-HIV-1 antibodies, exhibit a sensitivity of 98.3% ^12^ and 100% ^13^, respectively. The dual *in house* WB achieved individual sensitivities of 96.7% (58/60) for HTLV-1 and 98% (49/50) for HIV-1. Regarding specificity, Fujirebio, 99.0%-99.7% for HTLV-1 ^12^ and 96.1% for HIV-1 ^13^, the dual WB 100%. These similarity findings demonstrate that the analytical performance of our dual *in house* WB is highly comparable to that of both reference methods. Furthermore, the dual assay shows comparable performance to that of other manufacturers, MP Diagnostics ^20^, Biokit ^21^^,^^22^ and Biorad ^19^ (sensitivities: HTLV-1, 96.9%-100%; HIV-1, 94.9%-100%; specificities: HTLV-1, 89.2%-100%; HIV-1, 87%-100%) ^19^^-^^22^.

We did not identify cross-reaction or interferences in the group of interfering samples (false positives), which provides technical evidence in favor of the potential use of this assay as a confirmatory test ^15^^,^^17^. In addition, running the viral antigens individually by electrophoresis before their electrotransfer, avoids protein superposition, ensuring greater specificity and avoiding cross-reactions in the assay.

Individually, the panel of HTLV-1/HIV-1 positive coinfected individuals showed a 94.0% sensitivity (47/50; three indeterminate), compared with the multiple kit for three parasitoses which obtained 100% (20/20) ^16^. By not considering the three indeterminate results (6%) as false negatives, the actual diagnostic performance of both tests remains at maximum sensitivity levels.

With an overall sensitivity of 96.3% (154/160; six indeterminate) and an overall specificity of 100% (100/100), the dual *in house* WB qualifies as a test of high diagnostic accuracy. Compared to the use of individual kits, this dual *in house* WB test simplifies the analyst's operational workload and reduces costs ^12^^,^^13^, maintaining the same diagnostic efficacy ^16^.

In relation to diagnostic accuracy, the dual test yielded 100% repeatability and reproducibility. These results confirm that the assay operates stably under various environmental conditions of temperature, humidity, and altitude, being highly reliable and scalable for the network of laboratories in the country.

The main limitation of the study lies in its retrospective design based on samples from a serotheque. Although cryopreservation at -70 °C and reconfirmation using reference tests ensured the stability and integrity of biological components, it was not possible to prospectively evaluate the test's performance in individuals with active clinical diagnostic suspicion. On the other hand, the restrictive sample size per subcondition (n=5) of the interferents limited individual statistical analyses. Despite this, the consolidated sample (n_5_=50) guaranteed the evaluation objectives. In another limitation, HTLV-2 and HIV-2 retroviruses were not included in the dual *in house* WB; HTLV-2 because it is not endemic and rarely causes severe disease, and HIV-2 because no case has yet been reported in Peru.

It is concluded that, the dual *in house* WB, presents high diagnostic performance for the individual and simultaneous detection of anti-HTLV-1 and anti-HIV-1 antibodies. Given its low cost, it is positioned as a viable alternative for diagnostic confirmation in the network of regional reference laboratories. The development of multicenter and population-based field studies is suggested for an eventual scaling and implementation of the dual *in house* HTLV-1/HIV-1 WB test in public health programs.
