# Supplementary material for: Diagnostic performance of a dual western blot for the simultaneous detection of anti-HTLV-1 and anti-HIV-1 antibodies
Source: Rev Peru Med Exp Salud Publica. 2026 Sep 28;43(3):356–63. doi: 10.17843/rpmesp.2026.433.16741 (PMC13718571; doi:10.17843/rpmesp.2026.433.16741)
Supplement: Supplementary file 2 [file rpmesp-43-03-16741-s001.docx]

**MATERIAL SUPLEMENTARIO**

| **Grupos evaluados** | **Muestras referentes** | **Positivos WB dual *in house*** | **Negativos WB dual *in house*** | **Indeterminados WB dual *in house*** | **Sensibilidad (%)** | **Especificidad (%)** |  |
| --- | --- | --- | --- | --- | --- | --- | --- |
| **Positivos HTLV-1/VIH-1** | 50 | 47 | 0 | 3 | 94,0 | — |  |
| **Positivos HTLV-1** | 60 | 58 | 0 | 2 | 96,7 | — |  |
| **Positivos VIH-1** | 50 | 49 | 0 | 1 | 98,0 | — |  |
| **Negativos (aparentemente sanos)** | 50 | 0 | 50 | 0 | — | 100,0 |  |
| **Interferentes** | 50 | 0 | 50 | 0 | — | 100,0 |  |
|  |  |  |  |  |  |  |  |
|  |  |  |  |  |  |  |  |
| **Tabla S1.** Evaluación del desempeño diagnóstico del Western blot dual in house HTLV-1/VIH-1. (A) Distribución de resultados positivos, negativos e indeterminados obtenidos en los diferentes grupos de muestras y su estimación de sensibilidad y especificidad. (B) Parámetros generales de exactitud diagnóstica del ensayo, con sus IC 95%. | | | | | | |  |
|  |  |  |  |  |  |  |  |
|  |  |  |  |  |  |  |  |

**Figura S1.** Perfil proteico de cuatro lotes de antígenos de HTLV-1 en geles de poliacrilamida al 15% y coloreados con azul de Coomassie

HTLV-1

**Figura S2.** Perfil proteico de cuatro lotes de antígenos de VIH-1 en geles de poliacrilamida al 15% y coloreados con azul de Coomassie

VIH-1

**Figura 3S** Temperatura, humedad y altitud de los cinco laboratorios referenciales registrados en los ensayos al evaluar la reproducibilidad de la prueba Western blot dual *in house* HTLV-1/VIH-1
