# Supplementary material for: Diagnostic performance of a dual western blot for the simultaneous detection of anti-HTLV-1 and anti-HIV-1 antibodies
Source: Rev Peru Med Exp Salud Publica. 2026 Sep 28;43(3):356–63. doi: 10.17843/rpmesp.2026.433.16741 (PMC13718571; doi:10.17843/rpmesp.2026.433.16741)
Supplement: Translation

# Translation: Rendimiento diagnóstico de un *western blot* dual para la detección simultánea de anticuerpos anti-HTLV-1 y anti-VIH-1

**Authors:** Eduardo Miranda-Ulloa, Soledad Romero-Ruiz, Ronal Briceño-Espinoza, Andrés Vásquez-Domínguez, Dilan Suárez-Agüero

**Affiliations:** 1 Laboratorio de Referencia Nacional de Virus de Transmisión Sexual, Centro Nacional de Salud Pública, Instituto Nacional de Salud, Lima, Perú.

**Keywords:** Western Blot, HTLV-1, VIH-1, Diagnóstico, Sensibilidad y Especificidad

## Abstract

Esta investigación planteó determinar el rendimiento de un *western blot* (WB) dual *in house* para la detección individual y simultánea de anticuerpos anti-HTLV-1 y anti-VIH-1; la tira se diseñó con antígeno HTLV-1 (1,67 ug/mm) arriba y VIH-1 (1,34 ug/mm) abajo. Se evaluó el WB dual *in house* con 260 sueros referentes procedentes de una seroteca: 50 HTLV-1/VIH-1 positivos, 50 VIH-1 positivos, 60 HTLV-1 positivos, 50 negativos y 50 interferentes. La precisión se evaluó en cinco regiones del Perú. Se obtuvo una sensibilidad y especificidad general de 96,3% (IC 95%: 93,0‒99,5) y 100% (IC 95%: 99,5‒100,0), respectivamente. Individualmente, la sensibilidad fue del 94% en coinfectados HTLV-1/VIH-1, 96,7% en HTLV-1 y 98% en VIH-1. La repetibilidad y reproducibilidad fue del 100% en condiciones de 34‒3256 m s. n. m., 16,4‒37,9 °C y 42‒95% de humedad relativa. Concluimos que el WB dual *in house* HTLV-1/VIH-1 presenta alto rendimiento diagnóstico.

## INTRODUCCIÓN

El virus linfotrópico T humano tipo 1 (HTLV-1) y el virus de la inmunodeficiencia humana tipo 1 (VIH-1) son los retrovirus más prevalentes en el mundo ^1^^,^^2^. La Organización Mundial de la Salud (OMS) reconoce vacíos epidemiológicos críticos en torno al HTLV-1, aunque estudios previos estimaban que en Perú existen al menos 450 000 personas infectadas con HTLV-1. La OMS enfatiza la urgente necesidad de implementar una vigilancia epidemiológica activa para precisar la verdadera carga global de la enfermedad ^1^; por su parte, entre 2021-2025, el Ministerio de Salud (MINSA) de Perú notificó 46 975 nuevos casos de VIH-1 ^3^.

En Perú, las personas viviendo con VIH, reciben gratuitamente monitoreo y tratamiento antirretroviral, minimizando el riesgo de transmisión sexual y vertical ^4^, mientras que las personas portadoras del HTLV-1 no gozan de ese beneficio, por la falta de programas sistemáticos de tamizaje y seguimiento clínico. Implementar una estrategia similar en el Perú es una necesidad urgente, ya que, ante la falta de un tratamiento antirretroviral efectivo, el tamizaje materno y la consiguiente regulación de la lactancia materna constituyen la única ventana de oportunidad efectiva para cortar la cadena de transmisión intergeneracional de esta enfermedad de alto costo e impacto social ^(^^1^.

Existen investigaciones que han descrito prevalencias de 36,4% (1250/3431) de HTLV-1 en familiares de casos índices ^5^ y del grave impacto clínico reflejado en un hospital peruano que diagnosticó cáncer en 1934 portadores del virus ^6^. Al igual que otros países, el Perú, previene la transmisión sanguínea del HTLV-1 en sus bancos de sangre ^1^, pero sólo Brasil ^7^ y Chile ^8^ tamizan a sus gestantes y puérperas en el control prenatal para prevenir la transmisión materno-infantil ^7^^,^^8^, cuya tasa oscila entre 3,9% al 27% ^1^.

El HTLV-1 y el VIH-1 tienen factores de riesgo semejantes y vías de transmisión compartidas. En la poblacion peruana se ha reportado que entre 10% y 18,6 % de las personas infectadas con VIH-1 presentan coinfección con HTLV-1 ^9^^,^^10^. Esta coinfección se ha asociado con un mayor riesgo de muerte y una menor supervivencia en comparación con la infección únicamente por VIH-1, lo que subraya la importancia del diagnóstico de HTLV-1 en personas con VIH-1 ^11^.

En el diagnóstico serológico, el *inmunoblot* constituye el ensayo de referencia para la confirmación de VIH y HTLV. Actualmente, las pruebas se comercializan de manera independiente para cada patógeno ^12^^,^^13^, lo que conlleva a realizar múltiples ensayos cuando se requiere evaluar coinfecciones ^9^^-^^11^, incrementando el tiempo, los costos y la complejidad operativa. En ese contexto, según nuestro conocimiento, hasta la fecha, no existe a nivel internacional un sistema comercial que permita la confirmación simultanea de ambos retrovirus. Ante esto, la innovación propuesta consiste en el desarrollo de una prueba de *western blot* de diagnóstico dual basado en una tira que contenga, de forma inmovilizada, antígenos inmunogénicos de VIH-1 y HTLV-1. El uso de esta única tira permitirá la confirmación y diferenciación simultánea de ambos patógenos. El desarrollo de una prueba diagnóstica dual podría contribuir a establecer nuevos algoritmos diagnósticos, reducir los costos operativos y facilitar la vigilancia epidemiológica de ambos retrovirus en poblaciones clave y otros grupos de riesgo.

Por lo descrito, el presente estudio tuvo como objetivo determinar el rendimiento diagnóstico de un *western blot* (WB) dual *in house* para la detección individual y simultánea de anticuerpos anti-HTLV-1 y anti-VIH-1.

**MENSAJES CLAVE**

Motivación para realizar el estudio. La lactoferrina es un componente esencial de la leche materna con importantes funciones inmunológicas. Si bien, existen estudios previos sobre su variación según la etapa de lactancia, la evidencia en el contexto peruano es limitada.

Principales hallazgos. Se observó una mayor concentración de lactoferrina en las muestras de leche de transición en comparación con las muestras de leche madura.

Implicancias en salud pública. Estos hallazgos aportan información descriptiva sobre la concentración de lactoferrina en leche de transición y leche madura en población atendida en Lima Norte. La ausencia de muestras de calostro limita la caracterización completa de la variación de la lactoferrina desde las primeras etapas de la lactancia.

## EL ESTUDIO

Se aplicó un diseño transversal retrospectivo de evaluación de prueba diagnóstica durante el 2024-2025. El tamaño muestral fue calculado con una estimación independiente de la sensibilidad (esperada: 90%) y especificidad (esperada: 95%), aplicándose la fórmula de proporciones (confianza del 95%, Z=1,96; error admisible del 5%, W=0,05), siendo los tamaños mínimos calculados de 139 muestras positivas y 73 negativas.

Se realizó un muestreo no probabilístico por conveniencia a partir de la seroteca del Instituto Nacional de Salud de Perú, consolidando una muestra final de 260 sueros (160 positivos y 100 negativos) que superó holgadamente los límites mínimos exigidos.

Siguiendo las directrices de la guía STARD, se implementó un diseño de validación estructurado en cinco grupos de muestras de suero. Esta estrategia permitió evaluar la sensibilidad dual e individual mediante grupos de muestras positivas para HTLV-1/VIH-1, HTLV-1 y VIH-1, así como la especificidad, utilizando grupos de muestras de individuos aparentemente sanos y muestras con interferentes. Los criterios de inclusión exigieron un volumen mínimo de suero de 600 µL y la ausencia de evidencia macroscópica de hemólisis, lipemia o contaminación microbiana. Por el contrario, se excluyeron aquellas muestras con volumen insuficiente, sin código de identificación o carentes de un resultado previo que permitiera clasificar su condición.

La distribución de las 260 muestras en cinco grupos de muestras consistió en: coinfectados positivos HTLV-1/VIH-1 (n_1_=50); positivos VIH-1 (n_2_=50); positivos HTLV-1 (n_3_=60); negativos HTLV-1/2 de personas aparentemente sanas (n_4_=50); interferentes (n_5_=50), con otras condiciones e infecciones: dengue (5), hepatitis B (5), *Strongyloides* (5), tuberculosis (5), uveítis (5), tiroiditis (5), gestantes (5), enfermedad reumatoide (5), linfoma (5) y paraplejia espástica (5).

Se realizó la caracterización serológica utilizando como estándar de referencia (Gold Standard) las pruebas comerciales de Inmunoblot INNO-LIA™ HTLV I/II ^12^ e INNO-LIA™ VIH-1/2 ^13^^)^ de la marca Fujirebio, fabricadas en Bélgica, las cuales son metodologías de confirmación validadas globalmente. Las 260 muestras de suero fueron procesadas por ambas pruebas de referencia.

Para garantizar el cegamiento de las pruebas en los cinco grupos, las muestras se codificaron de forma aleatoria antes de ser procesadas con los dos estándares de referencia. Posteriormente a la obtención de sus resultados, se seleccionaron aquellas que cumplían con los criterios establecidos para cada grupo.

El grupo de interferentes se incluyó con el propósito de evaluar la reactividad cruzada y la interferencia analítica general, siendo ambas abordadas de manera conjunta y aunque el tamaño de la muestra se limitó a cinco muestras por subcondición, lo que restringe el análisis estadístico individual por subgrupos, el tamaño muestral consolidado (n_5_=50) otorgó la potencia estadística adecuada.

Las muestras de personas aparentemente sanas, fueron colectadas originalmente de donantes de banco de sangre que resultaron negativos en las pruebas de tamizaje para los siete marcadores obligatorios: VIH-1/2, HTLV-1/2, VHB (antígeno de superficie y anticuerpo core total), VHC, Chagas y sífilis.

Para la preparación de los antígenos HTLV-1 y VIH-1, se cultivó *in vitro* las líneas celulares MT-2+HTLV-1 y H9+HIVIIIB, siguiendo el procedimiento descrito por Romero-Ruiz *et tal*^14^; las concentraciones de los pellets celulares HTLV-1 tuvieron entre 30.050.000 y 41.000.000 de células, para VIH-1 tuvieron entre 25.250.000 y 45.350.000 de células. Cada pellet celular fue lavado, centrifugado, lisado, cuantificado y tratados los antígenos conforme al procedimiento detallado por Miranda-Ulloa *et al*^15^; el rango de concentraciones de las proteínas HTLV-1 estuvo entre 0,31 a 0,56 ug/ul, y de VIH-1 entre 0,23 a 0,52 ug/ul. Se prepararon cuatro lotes de antígenos de cada virus, caracterizándose sus proteínas en geles de poliacrilamida y tiñéndose con azul de Coomassie (material suplementario figura S1 y S2).

El WB múltiple para parásitos desarrollado por Davelois *et al.*^16^^)^ impulsó la creación de la metodología dual, tomándose como punto de partida las metodologías individuales desarrolladas por nuestro equipo investigador ^15^^,^^17^ con ciertas modificaciones, finalmente, se realizó el proceso estandarizado con los siguientes pasos:

Los antígenos tratados de cada virus fueron sometidos a electroforesis de manera individual en geles distintos, usándose minigeles de 8,3 × 7 × 0,1 cm del Mini PROTEAN® Tetra Cell-Bio Rad Laboratories, con un gel de resolución al 15% y un gel concentrador al 10%. El sistema del gel tuvo tres carriles, uno de 60 mm y los otros de 5 mm ubicados a los lados extremos. Los antígenos fueron sembrados en el carril de 60 mm con 1,67 ug/mm para HTLV-1 y 1,34 ug/mm para VIH-1. En los carriles laterales extremos se esparció 2 µL de marcador pre-teñido por carril. Los pasos subsiguientes se realizaron conforme a la metodología establecida por Miranda-Ulloa *et al.*^15^.

Se realizó una electrotransferencia húmeda en el Trans Blot Transfer Cell; el diseño consistió en ubicar sobre la parte superior de la membrana de nitrocelulosa de 0,22 um, el gel HTLV-1 y en la inferior el gel VIH-1, seleccionando previamente los antígenos específicos con ayuda del marcador pre-teñido, el cual facilitó delimitar las zonas de los geles a cortar. Los pasos posteriores hasta el bloqueo de la membrana y corte en tiras, se realizaron conforme al procedimiento detallado por Miranda-Ulloa *et al.*^15^.

Se realizó las corridas de los cinco grupos de muestras de suero usando placas de ocho canales, siendo el volumen óptimo para todos los pasos de 1 ml; cada tira fue sumergida en PBS-T-L conteniendo los sueros en estudio y controles a la dilución 1/20 por 3 horas en agitación a 90 rpm. Los pasos que continuaron se realizaron según el procedimiento detallado por Miranda-Ulloa *et al.*^15^.

Se incluyó en cada corrida, un control positivo y un negativo, los cuales consistieron en sueros caracterizados por las pruebas de referencia y validados por la presencia o ausencia total de bandas diagnósticas para HTLV-1/VIH-1, respectivamente.

Los criterios de lectura del WB dual *in house,* se realizaron de la siguiente manera: criterio, OMS: positivo HTLV-1: reactividad con al menos una proteína Env (gp46 o gp21) y al menos una proteína de núcleo (p19 o p24) ^18^. Criterio, Consorcio de la Red de Serologías: positivo VIH-1: reactividad con la proteína Env gp41 y al menos una proteína gag o pol, p55, p39, p31, p24 o p17 ^19^. Negativo: sin ninguna banda específica. Indeterminado: presencia de una sola banda, o patrones incompletos.

Con los resultados de los cinco grupos de muestras, se determinó la exactitud diagnóstica del WB dual *in house*. Los resultados indeterminados fueron considerados como no positivo (negativo) en la tabla de contingencia al calcular las respectivas sensibilidades. Los datos de la prueba de referencia y del WB dual *in house* se consolidaron en Microsoft Excel®. La validez diagnóstica se evaluó en Epidat v3.1 (módulo de pruebas diagnósticas simples) mediante tablas de contingencia de 2x2. Se calcularon la sensibilidad, especificidad, índice de validez, VPP, VPN, índice de Youden y razones de verosimilitud (positiva y negativa), todos con intervalos de confianza del 95% (IC 95%). La concordancia (%) de la precisión diagnóstica se evaluó utilizando cinco sueros de referencia (un suero de cada grupo de muestras); para determinar la repetibilidad, estos sueros se analizaron por quintuplicado en una sola corrida intralaboratorio y la reproducibilidad se calculó analizándolos por triplicado durante tres días diferentes en cinco escenarios geográficos distintos interlaboratorio (La Libertad, Junín, Apurímac, San Martín y Amazonas). Este diseño permitió verificar el desempeño de la prueba bajo diversas condiciones ambientales de temperatura, humedad y altitud, un factor crítico para asegurar su viabilidad en un escenario de descentralización.

Los sueros utilizados fueron anonimizados. El estudio fue aprobado por el Comité Institucional de Ética en Investigación del Instituto Nacional de Salud (OI-012-19) y financiado por PROCIENCIA - CONCYTEC (contrato N° 022-2019-FONDECYT).

## HALLAZGOS

Las 260 muestras fueron consideradas en el estudio y no se llegó a excluir ninguna, el flujograma presenta la distribución de los sueros incorporados según el estado serológico de referencia versus los resultados obtenidos mediante el WB dual *in house* HTLV-1/VIH-1 (figura 1).

**Figura 1:** Flujograma de selección y evaluación de las muestras incluidas en el estudio.

^a^Interferentes: negativos a HTLV-1/VIH-1, pero con otras infecciones y condiciones; dengue (5), hepatitis B (5), Strongyloides (5), tuberculosis (5), uveítis (5), tiroiditis (5), gestantes (5), enfermedad reumatoide (5), linfoma (5) y paraplejia espástica (5).

Se desarrolló un WB dual que detecta anticuerpos anti-IgG para HTLV-1 y VIH-1 simultáneamente en una sola tira. En las tiras correspondientes a los controles positivos duales e individuales, se visualizan según corresponda, las bandas características de ambos retrovirus (HTLV-1=p19, gp21, p24, gp46; VIH-1=p17, p24, p31, p39, gp41, p55), mientras que el control negativo, no mostró reactividad frente a ninguno de los sistemas antigénicos, validando el formato empleado (figura 2).

**Figura 2:** Patrón de reactividad de la prueba de *western blot* dual *in house* HTLV-1/VIH-1 para detección de anticuerpos en suero en controles internos del ensayo. CP1: HTLV-1 y VIH-1 positivos; CP2: VIH-1 positivo; CP3: HTLV-1 positivo; CN: control negativo.

PM: peso molecular, KD: kilodaltons

Para la evaluación de la exactitud diagnóstica, se determinó la sensibilidad individual del WB dual *in house* con las lecturas de cada panel positivo. Así, individualmente se obtuvo una sensibilidad de 94,0% (47/50) con el panel coinfectados HTLV-1/VIH-1 (figura 3A), 98,0% (49/50) con el panel VIH-1 (figura 3B) y 96,7% (58/60) con el panel HTLV-1 (figura 3C). Se obtuvo en cada uno de estos tres paneles positivos, tres, uno y dos resultados indeterminados, respectivamente (figura 1). La especificidad individual fue de 100% (50/50) con el panel negativo (figura 3D) y 100% (50/50) con el panel interferente (figura 3E) (material suplementario tabla S1).

**Figura 3:** Perfiles de bandas y evaluación de la exactitud y precisión diagnóstica del *western blot* dual in house HTLV-1/VIH-1. Los grupos de muestras de suero para la exactitud diagnóstica incluyeron: A) coinfección HTLV-1/VIH-1 positivos, B) VIH-1 positivos, C) HTLV-1 positivos, D) negativos, E) interferentes. La precisión del ensayo fue evaluada mediante los parámetros de F) repetibilidad y G) reproducibilidad.

Al analizar de manera conjunta los tres paneles positivos, se obtuvo una sensibilidad general de 96,3% (154/160) (IC 95%: 93,0-99,5), mientras que, al agrupar el panel negativo e interferente, se obtuvo una especificidad general de 100% (100/100) (IC 95%: 99,5-100,0). En conjunto, se obtuvo un índice de validez de 97,7%, con valores predictivos positivo y negativo de 100% y 94,3%, respectivamente. Asimismo, el índice de Youden fue de 0,96 y la razón de verosimilitud negativa 0,04 (tabla 1).

**Tabla 1:** Evaluación del desempeño diagnóstico del *western blot* dua*l in house* HTLV-1/VIH-1. Parámetros generales de exactitud diagnóstica del ensayo, con sus intervalos de confianza al 95% (IC 95%).

Parámetros	***Western blot* dual *in house* HTLV-1/VIH-1**		
	Valor	(IC 95%)	
Sensibilidad (%)	96,3	93,0	99,5
Especificidad (%)	100	99,5	100
Índice de validez (%)	97,7	95,7	99,7
Valor predictivo + (%)	100	99,7	100
Valor predictivo - (%)	94,3	89,5	99,2
Índice de Youden	0,96	0,93	0,99
Razón de verosimilitud +	-	-	
Razón de verosimilitud -	0,04	0,02	0,08

Con respecto a la evaluación de la precisión diagnóstica, el análisis de las cinco muestras procesadas por el WB dual *in house* en una sola corrida por quintuplicado en condiciones idénticas, mostraron perfiles de bandas concordantes, sin variaciones en la intensidad ni en la presencia de bandas diagnósticas, obteniendo una concordancia en la repetibilidad intralaboratorio del 100% (figura 3F). Del mismo modo, las cinco muestras analizadas por triplicado en cinco laboratorios referenciales con altitudes distintas de 34, 353, 420, 2378 y 3.256 m s.n.m., en un rango de temperaturas entre 16,4 °C y 37,9 °C y a una humedad relativa medioambiental entre 42% y 95% (material suplementario figura S3), dieron resultados concordantes obteniendo una reproducibilidad interlaboratorio, del 100% (figura 3G).

## DISCUSIÓN

Las pruebas de referencia inmunoblot Fujirebio (INNO-LIA) para la detección de anticuerpos anti-HTLV-1 y anti-VIH-1, exhiben una sensibilidad de 98,3% ^12^ y 100% ^13^, respectivamente. El WB dual *in house* alcanzó sensibilidades individuales de 96,7% (58/60) para HTLV-1 y 98% (49/50) para VIH-1. Respecto a la especificidad, Fujirebio, 99,0%-99,7% para HTLV-1 ^12^ y 96,1% para VIH-1 ^13^, el WB dual 100%. Estos hallazgos de similitud demuestran que el desempeño analítico de nuestro WB dual *in house* es altamente comparable al de ambos métodos de referencia. Además, el ensayo dual muestra un rendimiento comparable al de otros fabricantes, MP Diagnostics ^20^, Biokit ^21^^,^^22^ y Biorad ^19^ (sensibilidades: HTLV-1, 96,9%-100%; VIH-1, 94,9%-100%; especificidades: HTLV-1, 89,2%-100%; VIH-1, 87%-100%) ^19^^-^^22^.

No identificamos reacción cruzada ni interferencias en el grupo de muestras interferentes (falsos positivos), lo que aporta evidencia técnica a favor del uso potencial de este ensayo como prueba confirmatoria ^15^^,^^17^. Además, correr los antígenos virales individualmente mediante electroforesis antes de su electrotransferencia, evita la superposición de proteínas, garantizando mayor especificidad y evitando reacciones cruzadas en el ensayo.

Individualmente, el panel de coinfectados HTLV-1/VIH-1 positivos mostró un 94,0% de sensibilidad (47/50; tres indeterminados), en comparación con el kit múltiple para tres parasitosis que obtuvo un 100% (20/20) ^16^. Al no considerarse los tres resultados indeterminados (6%) como falsos negativos, el rendimiento diagnóstico real de ambas pruebas se mantiene en niveles de máxima sensibilidad.

Con una sensibilidad general del 96,3% (154/160; seis indeterminados) y una especificidad general del 100% (100/100), el WB dual *in house* califica como una prueba de alta exactitud diagnóstica. En comparación con el uso de kits individuales, esta prueba de WB dual *in house* simplifica la carga operativa del analista y disminuye los costos ^12^^,^^13^, manteniendo la misma eficacia diagnóstica ^16^.

En relación a la precisión diagnóstica, la prueba dual arrojó un 100% de repetibilidad y reproducibilidad. Estos resultados confirman que el ensayo opera de manera estable ante diversas condiciones medioambientales de temperatura, humedad y altitud, siendo altamente confiable y escalable para la red de laboratorios en el país.

La principal limitante del estudio radica en su diseño retrospectivo basado en muestras procedentes de una seroteca. Si bien la criopreservación a -70 °C y la reconfirmación mediante pruebas de referencia aseguraron la estabilidad e integridad de los componentes biológicos, no fue posible evaluar el desempeño de la prueba de forma prospectiva en individuos con sospecha diagnóstica clínica activa. Por otro lado, el tamaño muestral restrictivo por subcondición (n=5) de los interferentes, limitaron los análisis estadísticos individuales. Pese a ello, la muestra consolidada (n_5_=50) garantizó los objetivos de la evaluación. En otra limitante, no se incluyó en el WB dual *in house* a los retrovirus HTLV-2 y VIH-2; HTLV-2 por no ser endémico y porque rara vez genera enfermedad severa y VIH-2 por no reportase aún ningún caso en Perú.

Se concluye que, el WB dual *in house*, presenta un alto rendimiento diagnóstico para la detección individual y simultánea de anticuerpos anti-HTLV-1 y anti-VIH-1. Dado su bajo costo, se posiciona como una alternativa viable para la confirmación diagnóstica en la red de laboratorios de referencia regional. Se sugiere el desarrollo de estudios de campo multicéntricos y de base poblacional para un eventual escalamiento e implementación de la prueba de WB dual *in house* HTLV-1/VIH-1 en los programas de salud pública.
